# Effect of a povidone-iodine intrauterine infusion on progesterone levels and endometrial steroid receptor expression in mares

**DOI:** 10.1186/1751-0147-52-66

**Published:** 2010-12-16

**Authors:** Irene Kalpokas, Fernando Perdigón, Rodolfo Rivero, Marilina Talmon, Isabel Sartore, Carolina Viñoles

**Affiliations:** 1 Experimental Field nº1-Faculty of Veterinary Medicine-Uruguay; 2 Veterinary Laboratories Division (DILAVE) Northwest Regional Laboratory. Ministry of Livestock, Agriculture and Fisheries (MGAP), Paysandú, Uruguay; 3 Laboratory of Nuclear Techniques, Faculty of Veterinary Medicine, Montevideo, Uruguay; 4 National Research Institute for Agriculture (INIA)-Tacuarembó, Uruguay

## Abstract

**Background:**

Intrauterine infusions have been widely used for the treatment of endometritis in the mare. Nevertheless, their consequences on endocrine and endometrial molecular aspects are unknown. We studied the effect of a 1% povidone-iodine solution intrauterine infusion on progesterone levels, endometrial histology and estrogen (ERα) and progesterone (PR) receptor distribution by immunohistochemistry.

**Methods:**

Fourteen healthy mares were used in this study. Estruses were synchronized and seven mares were treated with intrauterine infusions at days 0 and 2 post ovulation of two consecutive estrous cycles. Uterine biopsy samples were taken on days 6 and 15 post ovulation.

**Results:**

The treatment did not induce an inflammatory response indicating endometritis, neither affected the ERα. However, it reduced the percentage of PR positive cells (PPC) on day 6 (deep glandular epithelium, control: 95.7 vs. infused: 61.5, *P *< 0.05). Treated mares tended to have lower progesterone levels on day 2 (3.9 ng/ml vs. 6.6 ng/ml, *P *= 0.07), and higher levels on day 15 compared with controls (4.4 ng/ml vs. 1.3 ng/ml, *P *= 0.07).

**Conclusion:**

a 1% povidone-iodine infusion during days 0 and 2 post ovulation in healthy mares did not induce histological changes indicating endometritis, but altered progesterone concentrations and reduced the expression of endometrial PR at day 6 without affecting the ERα. These changes could reduce embryo survival.

## Background

Endometritis is a major cause of infertility in the mare [1] and factors such as perineal conformation and uterine clearance that depend on the breed, age and reproductive status contribute to the pathogenesis [2]. Rapid physical clearance of uterine contents after mating or foaling is most important in the uterine defence [2]. Therefore, intrauterine infusions have been used widely in the equine practice as a treatment to clear the uterus within 96 hours post ovulation [2-4]. The objective is that the embryo encounters a healthy endometrium around day 6, when it enters the uterus [5]. Povidone-iodine solutions are often used for intrauterine infusions due to their low cost, easy of preparation, storage and delivery and especially because they are indicated for the treatment of fungal infections [2,4]. However, contradictory findings have been reported on the consequences of using this antiseptic. A 0.05% povidone iodine solution infused into the uterus around the time of ovulation did not result in an inflammatory reaction on day 6 post ovulation [3] and did not affect pregnancy rates [6]. On the other hand, histopathological findings reported by Olsen et al. [7] led to the conclusion that a 1% povidone-iodine intrauterine solution generates acute and chronic inflammatory changes in the endometrium. Nevertheless, all these experiments were carried out with mares of different (or even unspecified) breed, age, and reproductive status, all factors that can clearly influence endometrial responsiveness to treatment [2,8].

The mare's endometrium is composed of various cell types (luminal and glandular epithelia, stromal cells, vascular cells) that undergo cyclical variation in their structure and function [9,10]. Both estradiol and progesterone mediate these changes and, should the mare conceive, prepare the uterine environment for the embryo's arrival and subsequent development [11]. These actions are mediated through specific intracellular receptors, namely, the alpha estrogen receptor (ERα) and the progesterone receptor (PR) [12], whose spatial expression on the day of embryo arrival to the uterus and on the day of luteolysis are critical for the establishment of pregnancy [11]. The expression of endometrial ERα and PR in the mare is closely related to the peripheral plasma hormone concentrations. Rising estradiol during proestrus and estrus induce synchronous expression of ERα and PR on stromal cells. Maximum progesterone values during early diestrus (day 5) are associated with the highest hormone receptor expression in epithelial cells [9,13]. Therefore, a decrease in the circulating concentrations of progesterone induced by the release of prostaglandins during an acute inflammatory process [14,15] may alter the expression of the sex steroid receptors in the endometrium [11].

The consequences of povidone-iodine infusions on endocrine and endometrial molecular aspects have not been described. If the treatment impairs endometrial steroid receptor expression, both directly (inflammation) [9,16] or indirectly (affecting hormone levels) [11], fertility could be compromised as it might cause failure of embryonic growth and/or maternal recognition of pregnancy [17]. These features need to be studied in young healthy mares, without the influence of factors that increase their susceptibility to an endometritis [2]. Our hypothesis was that a povidone-iodine infusion would induce an endometrial inflammatory reaction that will decrease progesterone concentrations and the expression of sex steroid endometrial receptors.

The aim of this study was to evaluate the effect of an intrauterine treatment with 1% povidone-iodine on plasma progesterone concentrations, endometrial histology and ERα and PR expression on the expected day of entry of the embryo into the uterus (day 6) and the onset of luteolysis (day 15).

## Methods

### Animals and treatments

This experiment was conducted during the breeding season at the Experimental Farm number 1 of the Veterinary Faculty, University of Uruguay, after approval by the Bioethics Committee of the same institution.

Fourteen Uruguayan Criollo Horse cross-breed mares aged 4 to 7, with no history of fertility problems were checked for health status by physical and ultrasonographic examination (Omega Vision, Vision Scanners, EI Medical, Loveland, CO, USA), uterine cytology and culture of uterine fluids, as described by Blanchard et al. [18]. The mares were aseptically prepared and sterile equipment used throughout the experiment. All mares were negative in bacteriological and cytological examinations.

To synchronize estrus, mares were given two injections of cloprostenol (250 μg i.m.) (Estrumate^®, ^Schering-Plough Animal Health Friesoythe Essex, Germany) 14 days apart. All mares were teased with a stallion and follicular development was monitored daily by transrectal ultrasonography. Once the dominant follicle reached 35 mm in diameter, mares were given 2500 IU of hCG i.m. (Chorulon^®^, Intervet International BV, Netherlands). Follicular monitoring continued until ovulation (day 0).

Seven mares (infused group) were treated with intrauterine infusions on days 0 and 2 using 1000 ml of a 1% povidone-iodine solution, as described by Olsen et al. [7]. The uterus was massaged gently to distribute the solution, which was left in situ.

Endometrial biopsy samples were collected [10] in all mares on day 15 of the estrous cycle. Synchronization was repeated in the next cycle and the same seven mares were treated with intrauterine infusions on days 0 and 2 post ovulation, but biopsy samples were taken on day 6 in both groups. Biopsies were collected in different cycles in order to avoid luteolysis. To check the presence of a single corpus luteum (CL), ultrasound examinations were performed in all mares on both days of biopsy collections (6 and 15 post ovulation).

Samples were fixed in 4% paraformaldehyde until assay.

### Hormone Determination

Blood samples were collected on days 0, 2, 6, 9 and 15 and 0, 2 and 6 of each cycle, respectively. Progesterone concentrations were measured by radioimmunoassay. Sensitivity was 0.13 ng/ml for low (0.6 ng/ml), medium (1.4 ng/ml) and high (5.7 ng/ml) control samples, the intra-assay coefficients of variation (CV) were 7.6%, 11.8% and 6,1%, respectively, whereas the inter-assay CV were 12.7% (0.75 ng/ml), 6.8% (1.9 ng/ml) and 7.4% (8.5 ng/ml), respectively.

### Receptor protein localization and abundance

Avidin-biotin-peroxidase immunohistochemical technique was used to visualize ERα and PR immunostaining [19]. Paraffin sections were cut (5 μm). We used mouse monoclonal antibodies to visualize ERα and PR (ERα: C-311, cat # sc-787, Santa Cruz, California, USA; PR: Zymed cat # 18-0172, South San Francisco, CA, USA, respectively) at different dilutions (ERα 1:25, PR 1:100). Negative controls were obtained by replacing the primary antibody with non-immune serum at similar concentrations. After primary antibody exposure, sections were incubated with biotinylated horse anti mouse IgG (Vectastain, Vector Laboratories) diluted 1:200 in normal horse serum. Sections were incubated with avidin-biotin complex peroxidase (Vectastain Elite, Vector Laboratories). The location of the bound enzyme was visualized by 3, 3' diaminobenzidine in H2O2 (DAB kit; Vector) and sections were counterstained with haematoxylin. For each receptor, all samples were analyzed in the same immunohistochemical assay.

### Image analysis

The amount of ERα and PR in different cell types was estimated subjectively by two independent observers; both receptors were evaluated in five endometrial compartments: luminal epithelium, glandular epithelium (arbitrarily divided in two sections, superficial and deep) and stroma (classified as superficial and deep following the same criteria). Ten fields were analyzed for each cell type at a magnification of × 1000 in all mares. The staining intensity was classified as negative, faint, moderate or intense and each category was expressed as a percentage of the total amount of cells. From this evaluation two variables were studied: the proportion of positive cells (PPC) and the staining intensity (SI). The staining intensity was calculated using the following formula: 1xn1+2xn2+3xn3, were n equals the proportion of cells with faint (1), moderate (2) or intense (3) staining [20].

### Histological analysis

Samples were fixed in paraformaldehyde and embedded in paraffin. Paraffin blocks were serially sectioned at 5 μm, stained with haematoxylin-eosin and evaluated according to the criteria of Kenney and Doig [21]. This included an evaluation of the stage of the estrous cycle and the pathological findings. Cellular infiltrations were evaluated based on the presence of different cell types (neutrophils, lymphocytes, eosinophils, macrophages and plasma cells), their distribution (lumen, stratum compactum, stratum spongiosum or within the glandular lumen), their frequency (average in linear fields of 5.5 mm, five per endometrial cellular section described above) and the severity of the inflammatory process. The severity of cellular infiltrations was subjectively graded as mild (a few cells in stroma), moderate (diffusely in the stratum compactum and/or frequently in the periglandular area or within the gland lumen) or severe (diffusely throughout the endometrium and frequently induced pleomorphism of the epithelium). The extent of the endometrial fibrosis was evaluated by calculating the number of periglandular fibrotic layers, and was graded as absent, slight (1 to 3 cell layers), moderate (4 to 10 cell layers) or severe (11 or more layers). The number (average in linear fields of 5.5 mm) and pattern of distribution of fibrotic nests were evaluated, as well as any associated glandular atrophy and/or cystic glandular change. Also hypertrophy, nonseasonal endometrial hypoplasia, and lymphatic lacunae were evaluated.

### Statistical analysis

All variables were subjected to analysis of variance using a mixed model (Statistical Analysis System; SAS Institute, Cary, NC, USA). The variables studied in the analysis of receptor localization by immunohistochemistry were the proportion of total positive cells and the staining intensity of the 10 fields. The statistical model included the effects of observer, treatment, day, cell type and section (luminal epithelium, superficial and deep glandular epithelium, and superficial and deep stroma) and their interactions. Progesterone levels were subjected to the same analysis; variables included day, treatment and their interactions. The level of significance was considered to be *P *< 0.05.

## Results

Results of the analysis of variance for progesterone levels and expression of steroid receptors are shown in Table 1. There was an effect of treatment on PR PPC (*P *< 0.05) and a highly significant effect of *treatment × day × section × cell type *interaction (*P *< 0.001).

**Table 1:** Level of significance of fixed effects and interactions studied in the statistical model.

Variable	**treat**.	day	treat. × day	**obs**.	cell type	**sect**.	**treat. × day × sect. × cell type**.
P4	NS	***	**				
ERα							
PPC	NS	*	NS	NS	***	**	NS
SI	NS	*	NS	NS	***	**	NS
PR							
PPC	*	NS	NS	0.1	***	***	***
SI	NS	NS	NS	*	***	***	***

NS: not significant

*P <0.05, ** *P <*0.01,

*** *P <*0.001

Fixed effects for progesterone (P4) are treatment (treat.: with or without infusion), day post ovulation (day: 0, 2, 6, 9, and 15) and their interactions. For estrogen and progesterone receptor (ERα; PR) are as well treatment, day (6 y 15), observer (obs.), cell type (luminal epithelium, glandular epithelium, stroma), section (sect.: superficial or deep) and their interactions. PPC, percentage of positive cells; SI, staining intensity.

### Histological analysis

Histopathological findings are shown in Table 2. In all sections, histological aspects were compatible with a physiologically active endometrium of cyclic mares in diestrus. The luminal epithelium of all mares was columnar and the height, glandular tortuosity and density increased in samples from day 15 compared to day 6, while stromal edema diminished. There were no signs of endometritis or endometriosis in any of the evaluated samples, which were classified as category I (there were either no abnormalities noted or only slight, widely scattered changes). Scarce isolated inflammatory cells (lymphocytes, neutrophils, macrophages, eosinophils) were observed in the uterine biopsy sections of a few mares from the control and infused group. The anatomical pattern was superficial, involving only the luminal epithelium and the stratum compactum. No focal areas of inflammation or other lesions (fibrosis, fibrotic nests, glandular atrophy and/or cysts, hypertrophy, hypoplasia, and/or lymphatic lacunae) were found.

**Table 2:** Histopathological findings and categorization of slides from mares on days 6 and 15 in the control group (a; b) and the infused group (c; d), respectively.

	Mare nº	Luminal epithelium	Stromal edema	Glandular tortuosity and density	Inflammatory Cells (type and frequency*)			
					**LC**	**MP**	**NP**	**EO**

a) Control group day 6 (n = 4)	545	height +	+	++				
	628	height +	+	++	0,2/field			
	5170	height +	+	++				
	5217	height +	+	++				

b) Control group day 15 (n = 4)	545	height ++	-	+++	0,2/field			
	628	height ++	-	+++		0,15/field		
	5170	height ++	-	+++				
	5217	height ++	-	+++				

c) Infused group day 6 (n = 6)	542	height +	+	++				
	632	height +	+	++				
	711	height +	+	++			0,4/field	0,3/field
	5135	height +	+	++		0,15/field	0,2/field	
	5172	height +	+	++				
	5178	height +	+	++				

d) Infused group day 15 (n = 7)	542	height ++	-	+++				
	632	height ++	-	+++				
	711	height ++	-	+++		0,1/field	0,2/field	
	5135	height ++	-	+++		0,15/field	0,2/field	
	5158	height ++	-	+++				
	5172	height ++	-	+++				
	5178	height ++	-	+++				

.(*) Cells were counted in 20 fields (x 400), 5 per endometrial cellular section and a mean number per field was calculated. LC, lymphocytes, MP, macrophages, NP, neutrophils, EO, eosinophils

### Progesterone levels

**Figure 1:**
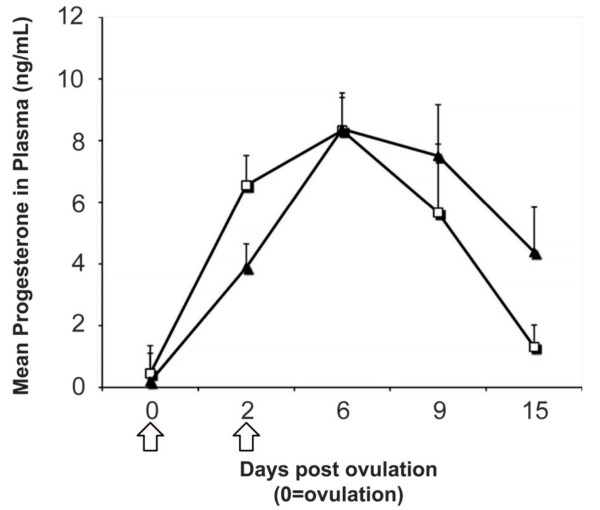
**Mean (± s.e.m) progesterone concentrations of control (open squares) and infused (solid triangles) mares**. Samples of consecutive cycles were pooled. Arrows indicate days of treatment of the infused group.

Progesterone reached maximum levels in early diestrus (on day 6) (Figure 1), lower concentrations were observed on the 9th day and continued to decline in late diestrus. Concentrations were affected by the *treatment × day *interaction (*P *< 0.01). Infused mares showed a tendency for lower progesterone levels on day 2 (3.9 ± 0.8 vs. 6.6 ± 0.9 ng/ml, *P *= 0.07), and higher levels on day 15 compared with controls (4.4 ± 1.4 vs.1.3 ± 0.7 ng/ml, *P *= 0.07).

### Receptors

**Figure 2:**
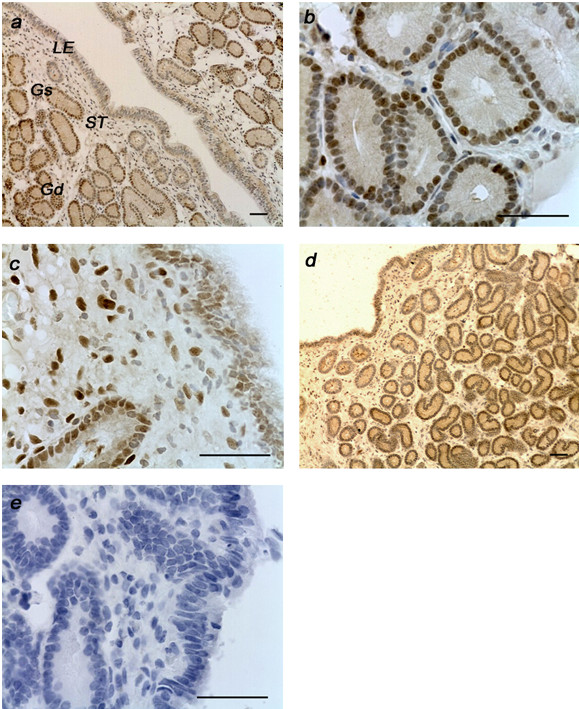
**Immunohistochemical localisation of estrogen receptor α in representative endometrial cross sections of mares on days 6 and 15 in the control group (a; c) and the infused group (b; d), respectively**. (e = negative). Original magnification × 100 and × 400. Scale bars = 100 μm. Gs, superficial glandular epithelium; Gd, deep glandular epithelium; LE, luminal epithelium; ST, stroma.

**Figure 3:**
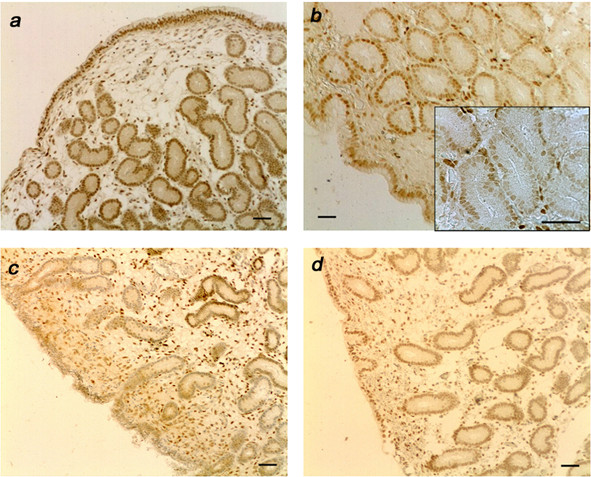
**Immunohistochemical localisation of progesterone receptor in representative endometrial cross sections of mares on day 6 and 15 in the control group (a; c) and the infused group (b; d), respectively**. Original magnification × 100 and × 400. Scale bars = 100 μm.

**Figure 4:**
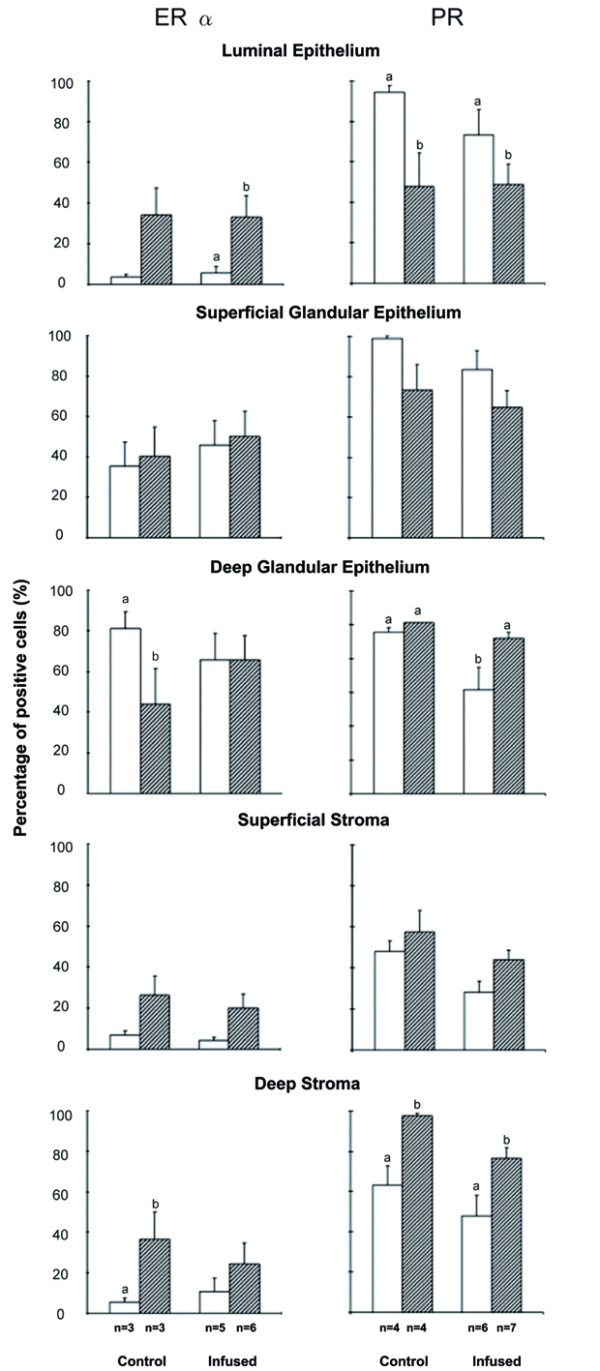
**Percentage of positive cells of estrogen receptor α (ERα; left panel) and progesterone receptor (PR; right panel) in control and infused mares on days 6 (open bars) and 15 (solid bars) post ovulation**. Bars with different superscripts within the same graph differ: a, b *P <*0.05.

Immunoreactive ERα and PR were detected exclusively in the nuclei of all endometrial cell types. When specific monoclonal antibodies were substituted with a non-immune mouse IgG, the absence of staining confirmed the high specificity of immunostaining for both receptors (Figure 2). Details of the changes in staining for the different days and treatments are shown in Figure 2, 3 and 4. Estrogen and progesterone receptor expression varied among days in most cell types and the glandular epithelium had the highest PPC for both receptors on days 6 and 15 (Figure 4). The treatment reduced PR PPC on day 6 at the deep glandular epithelium level (*P <*0.05) (Figure 4).

### Estrogen receptor

ERα immunostaining was mild to moderate, with more staining on day 15 compared with day 6 (*P *< 0.05) in all cell types except in the deep glandular epithelium, where staining was greater on day 6 (*P *= 0.08), and levels were similar in the infused group. The PPC and SI for ERα were affected also by cell type (*P *< 0.001), with more staining in the glandular epithelium. Povidone-iodine infusion did not affect the expression of this receptor (Table 1).

### Progesterone receptor

A higher immunoreactivity was found in the luminal epithelium (PPC: *P *< 0.001; SI: *P *< 0.01) and superficial glandular epithelium (*P *= 0.08) on d 6 compared with day 15, with an opposite pattern in deep stroma (*P *< 0.01). Povidone-iodine treated mares had lower PPC for PR (*P *< 0.05) but the treatment did not affect SI. Nevertheless, there was a highly significant cell type and *treatment × day × cell type × section *interaction effect both for PPC and SI (Table 1). A reduction in receptor levels was seen on day 6 in the infused group compared with controls (Figure 3 and 4), and PR significantly decreased in the deep glandular epithelium in the infused group (Table 1 Figure 4).

## Discussion

This is the first study that shows that a povidone-iodine intrauterine infusion affects progesterone levels and steroid receptors in the equine endometrium. The hypothesis that a 1% povidone-iodine infusion would induce an long term inflammatory response in the endometrium was rejected. However, the treatment reduced PR expression and progesterone levels during the early luteal phase.

There were no histological signs of endometritis or endometriosis, based on the absence of abnormalities (e.g. fibrosis, hypertrophy) and the scarce presence of neutrophils, considered the "best standard" for diagnosing acute endometritis [22]. Although some inflammatory cells (neutrophils, macrophages, eosinophils) were observed in some mares, this probably correspond to a mild inflammatory cellular reaction following the biopsy [7], and the normal dynamic populations of leucocytes present in this tissue [23]. We infused a greater volume of solution than Olsen et al., [7] to reach the entire endometrial surface, but histological signs of endometritis were not observed, at least not on day 6. If the uterus started to recover soon after the infusion, the sampling may have been too late to diagnose pathological alterations. Fumuso et al. [23] reported that in healthy mares, an inflammatory stimulus resulting in increased immune cells during estrus was solved by day 7. Adverse effects depend also on the resistance of the mare [2,8], and we used young healthy mares. However, our results agree with those of Brinsko et al. [3] who conclude that a 0.05% povidone-iodine infusion at ovulation does not generate inflammatory changes at day 6. To study and compare the histological and molecular effects of different povidone-iodine solutions concentrations could provide valuable information when deciding the therapeutic option in the equine practice.

Progesterone profiles observed in the control group were consistent with those described by Nagy et al. [24]. Hormone levels were affected by the infusion, as there was a *treatment × day *interaction (*P *< 0.01) and a tendency to lower progesterone levels in the infused group on day 2 (*P *= 0.07). Since there were no histological signs of inflammation, we suggest that this could be explained by a transient release of prostaglandins triggered by the cervical manipulation or the intra uterine infusion that delayed CL development [25]. Moreover, Troedsson et al. [26] concluded that repeated injections of a prostaglandin analogue within the first 48 hours post ovulation affected luteal function in a reversible and temporary manner. This may also be the case in our study, although a more frequent sampling protocol to measure progesterone might have improved the interpretation of our data. Moreover, Troedsson et al. [26] clearly demonstrated the importance of high progesterone levels during the early luteal phase, since temporal decreases in progesterone concentrations resulted in lower pregnancy rates in the treated mares (12.5%) compared with controls (62.5%).

The most important observation was that the infusion caused a decrease in the PR immunostaining (effect on PPC), with a significant effect of *treatment × day × section × cell type *interaction. Progesterone actions, mediated through the PR, are critical in preparing the endometrium for pregnancy. The increase in progesterone concentrations post-ovulation elicits the proliferation and maximal ERα and PR expression in the luminal and glandular epithelium [9], in addition to the formation of functional glands. Endometrial glands synthesize, secrete and transport histotroph, which is essential for the survival of the equine conceptus [27]. The functional asynchrony of epithelial cells is associated with subfertility in the mare [16]. In our study, there seemed to be an overall decreased PR expression in glandular epithelial cells and in stromal cells on both days of evaluation in infused mares. The decrease was significant in the glandular epithelium on day 6, the day of expected arrival of the embryo to the uterus. If the treatment decreased the sensitivity of the glandular endometrium to progesterone, then embryo development and subsequently maternal recognition of pregnancy may be altered in treated mares, as is the case for ewes [28]. The inability of the endometrium to respond adequately to steroid hormonal stimuli may represent one of the causes of subfertility in mares [29]. The treatment did not affect SI of RP; although the difference may be related to the greater subjectivity of SI evaluation compared to PPC, it also reveals the complexity of the mechanisms and signalling pathways involved in receptor expression [12].

Although there was a significant *day × treatment *effect, no differences in progesterone levels were detected at day 6, when less PR was found in the endometrium. These results suggest that circulating progesterone may have not been responsible for the down regulation of PR. However, the lower progesterone levels in the infused group on day 2 could have affected PR on day 6 if we consider that the process of gene transcription and mRNA translation is not likely to induce measurable effects within the cell or tissue until hours or even days after steroid stimulation [12]. On the other hand, many studies have revealed several alternative receptor-signalling mechanisms that diverge from the classic model [12]. Nonetheless, due to our experimental design we cannot conclude, whether povidone-iodine, prostaglandin or some other factor present in the infused group act through an alternative pathway, therefore further research is necessary to investigate this hypothesis.

The infusion did not have an effect on the ERα, but it should be noted that the results for this receptor are limited due to the small number of samples evaluated in the control group. Although a selective effect of certain toxics (i.e. dioxins) on PR has been reported in humans [30], we cannot assume that based on the results of our study.

The sensitivity of epithelial and stromal cells to the sex steroids was different on days 6 and 15 post ovulation. The expression of PR decreased in the luminal and superficial glandular epithelium and increased in the superficial and deep stroma in both groups of mares on day 15. Our findings support previous reports that clearly indicate that the loss of PR in uterine epithelia is normal during the course of the estrous cycle and a prerequisite for implantation in pregnant mammals [11,31]. Another expression pattern of PR was seen on day 15 in the deep glandular epithelium: in control mares immunostaining remained similar to day 6 and treated mares reached similar levels of those of the control group, which may indicate an endometrial recovering capacity [24]. Nevertheless, it has been suggested that decreases in uterine sex steroid gene expression during the first week of gestation may be sufficient to cause pregnancy failure [17]. It can be argued that the infusion process per se may have promoted the disturbance in the PR expression that could not be tested since the control mares were not infused with saline. However, our findings are supported by those of Olsen et al. [7], who found no severe endometrial changes in mares infused with saline.

We conclude that a 1% povidone-iodine infusion on days 0 and 2 post ovulation in mares does not induce long-term histological signs of endometritis, but affects plasma progesterone concentrations and reduces endometrial PR expression on day 6 post ovulation without affecting ERα expression. These findings may provide valuable information for choosing the appropriate treatment and diagnostic method in equine practice.

## Abbreviations

CL: Corpus luteum; ERα: estrogen receptor alpha; IgG: immunoglobulin G; H202: hydrogen peroxide; mRNA: messenger Ribonucleic acid; PPC: percentage of positive cells; PR: progesterone receptor; SI: staining intensity.

## Competing interests

The authors declare that they have no competing interests.

## Authors' contributions

CV, FP, RR and IK conceived the experimental design and participated in the data collection. FP and IK checked the reproductive status of the mares. IK collected all samples (blood, uterine for cytology and biopsy); applied the synchronization treatments and performed the ultrasound evaluations of the ovaries; performed the povidone-iodine infusions; and was trained to analyse the progesterone by RIA. IS and IK performed the immunohistochemistry procedures. MT and IK evaluated the immunostainings for ERα and PR. IK performed the H&E staining and with RR evaluated the samples according to the criteria of Kenney and Doig. CV and IK performed the statistical analysis. All authors have been involved in drafting the manuscript and then read and approved the final manuscript.
